# Cholesterol Biosynthesis and Homeostasis in Regulation of the Cell Cycle

**DOI:** 10.1371/journal.pone.0058833

**Published:** 2013-03-15

**Authors:** Pushpendra Singh, Roopali Saxena, Gunda Srinivas, Gopal Pande, Amitabha Chattopadhyay

**Affiliations:** Centre for Cellular and Molecular Biology, Council of Scientific and Industrial Research, Hyderabad, India; Emory University School of Medicine, United States of America

## Abstract

The cell cycle is a ubiquitous, multi-step process that is essential for growth and proliferation of cells. The role of membrane lipids in cell cycle regulation is not explored well, although a large number of cytoplasmic and nuclear regulators have been identified. We focus in this work on the role of membrane cholesterol in cell cycle regulation. In particular, we have explored the stringency of the requirement of cholesterol in the regulation of cell cycle progression. For this purpose, we utilized distal and proximal inhibitors of cholesterol biosynthesis, and monitored their effect on cell cycle progression. We show that cholesterol content increases in S phase and inhibition of cholesterol biosynthesis results in cell cycle arrest in G1 phase under certain conditions. Interestingly, G1 arrest mediated by cholesterol biosynthesis inhibitors could be reversed upon metabolic replenishment of cholesterol. Importantly, our results show that the requirement of cholesterol for G1 to S transition is absolute, and even immediate biosynthetic precursors of cholesterol, differing with cholesterol merely in a double bond, could not replace cholesterol for reversing the cell cycle arrest. These results are useful in the context of diseases, such as cancer and Alzheimer’s disease, that are associated with impaired cholesterol biosynthesis and homeostasis.

## Introduction

**Figure 1:**
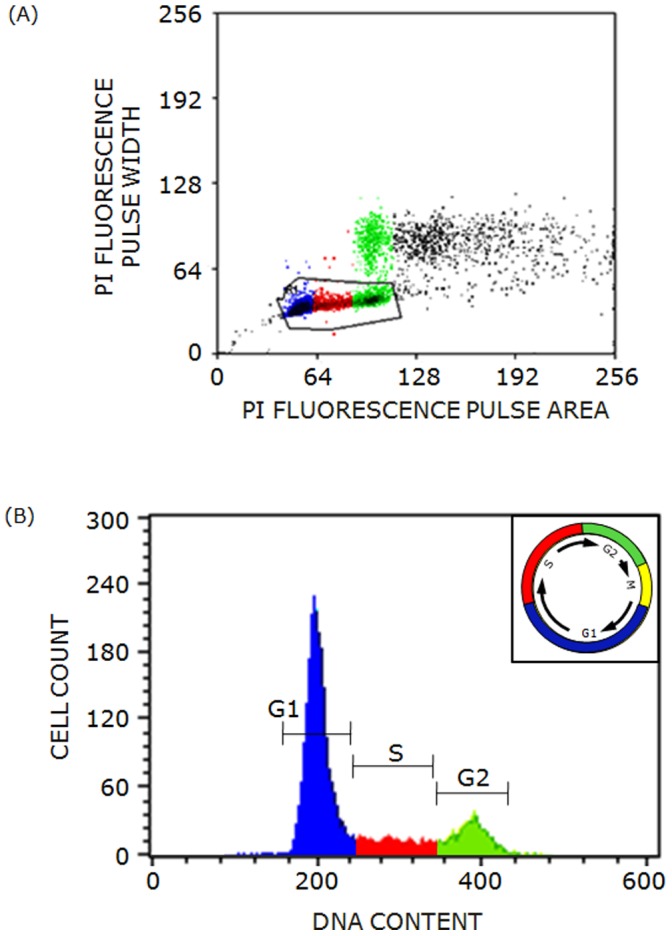
Flow cytometric analysis of asynchronous F111 cells. (A) Pulse width analysis of cells was carried out to discriminate between singlets and multiplets of cells. (B) Representative flow cytometric profile of asynchronous F111 cells was acquired upon propidium iodide labeling. The histogram depicts the distribution of cells in G1 (blue), S (red) and G2 (green) phases of the cell cycle. The inset shows a time-scaled diagram of different phases of cell cycle. See Materials and Methods for more details.

The cell cycle represents an ordered series of events that continuously occur in all living cells that comprise multicellular organisms and undergo multiplication. Non-multiplying cells are therefore often considered to be out-of-cycle or arrested in the cell cycle. Most cells multiply by mitotic division which is represented by the M phase in the cell cycle. The M phase is preceded and followed by successive G1, S and G2 phases (see Fig. 1A) and therefore it represents the culmination of one, and beginning of another cycle. G1 and G2 phases represent two ‘gaps’ that occur between mitosis and DNA synthesis, and between DNA synthesis and mitosis. Cells prepare for DNA synthesis in G1 phase, increase their DNA content from 2N to 4N in S phase and prepare for mitosis with double the normal DNA content per cell in G2 phase [1]. These phases of cell cycle can be identified on the basis of changes in cellular DNA content in a population using flow cytometry (shown in Fig. 1B). The progression and transition of cells between the phases of the cell cycle is tightly regulated and controlled by a series of checkpoints. A very large number of cytoplasmic and nuclear regulators of cell cycle have been identified, yet the role of cell membrane lipids in this process is unclear. For example cholesterol biosynthesis has been shown to be necessary for growth and division of mammalian cells [2]–[4] but its role in regulation of cell cycle progression is not yet clearly understood.

**Figure 2:**
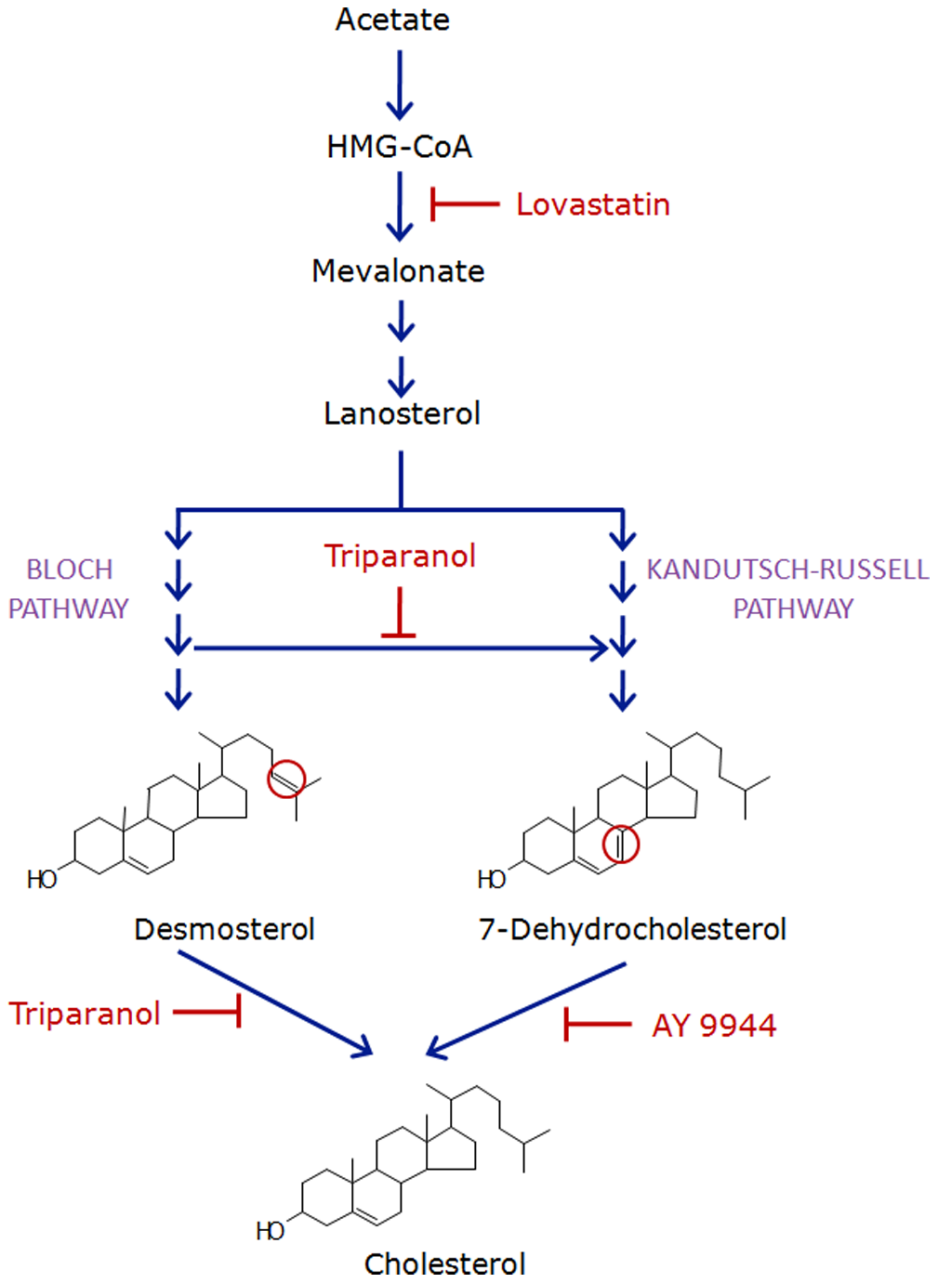
A schematic representation of various inhibitors that inhibit cholesterol biosynthesis. Cholesterol biosynthesis takes place by two pathways, namely, the Kandutsch-Russell and Bloch pathways. These pathways have common initial steps starting from acetate and branch out after lanosterol. Although these pathways do not have a common intermediate after lanosterol, they are linked by an enzyme called 24-DHCR. The only difference between equivalent sterol intermediates of the two pathways is the presence of double bond at the 24^th^ position in the alkyl side chain of sterol intermediates of the Bloch pathway. 24-DHCR catalyzes the reduction of the double bond at the 24^th^ position in the alkyl side chain of sterols. These reduced sterol intermediates are utilized at different steps of the Kandutsch-Russell pathway of cholesterol biosynthesis. The availability of inhibitors that block cholesterol biosynthesis at various steps has proven to be extremely useful in elucidating the role of cholesterol and its precursors in regulating various physiological processes. In order to probe the stringency of cholesterol requirement for cell cycle progression, we utilized proximal (lovastatin) and distal (triparanol and AY 9944) inhibitors of cholesterol biosynthesis. The key steps of cholesterol biosynthesis blocked by these inhibitors are shown in the figure. Statins are competitive inhibitors of HMG-CoA reductase, the key enzyme in cholesterol biosynthesis that catalyzes the conversion of HMG-CoA into mevalonate. Triparanol is a metabolic inhibitor of the enzyme 24-DHCR which catalyzes the conversion of desmosterol into cholesterol (the last step of the Bloch pathway) by reducing the unsaturation at the 24^th^ position of desmosterol. As mentioned above, 24-DHCR is essential for occurrence of the Kandutsch-Russell pathway. Triparanol treatment of cells therefore inhibits cholesterol biosynthesis *via* both the Kandutsch-Russell and the Bloch pathways. On the other hand, AY 9944 (metabolic inhibitor of 7-DHCR) specifically inhibits the last step of the Kandutsch-Russell pathway of cholesterol biosynthesis. See text for more details.

Cholesterol is an essential component of higher eukaryotic membranes and plays an important role in cell membrane organization, dynamics and function. It is the end product of a long, multi-step and exceedingly fine-tuned sterol biosynthetic pathway involving more than 20 enzymes. According to the ‘Bloch hypothesis’, the sterol biosynthetic pathway parallels sterol evolution. In other words, cholesterol biosynthetic pathway have evolved by the process of natural selection to optimize properties of eukaryotic cell membranes for specific biological functions [5]. Cholesterol biosynthesis in cells takes place by two pathways, namely, the Kandutsch-Russell and the Bloch pathway (see Fig. 2). These pathways have common initial steps starting from acetate and branch out at lanosterol. The first rate-determining enzyme in the cholesterol biosynthetic pathway is HMG-CoA reductase which catalyzes the conversion of HMG-CoA into mevalonate, and represents a common step for both pathways. Subsequently, mevalonate is utilized for both non-sterol isoprenoid and cholesterol biosynthesis. 7-dehydrocholesterol (7-DHC) and desmosterol are immediate biosynthetic precursors of cholesterol in the Kandutsch-Russell and Bloch pathways, respectively. 7-DHC differs with cholesterol only in an extra double bond at the 7^th^ position in the sterol ring [6]. Likewise, desmosterol has an extra double bond at the 24^th^ position in the flexible alkyl side chain of the sterol [7]. Importantly, 3β-hydroxy-steroid-Δ^7^-reductase (7-DHCR) catalyzes the conversion of 7-DHC to cholesterol in the last step of the Kandutsch-Russell pathway. On the other hand, 3β-hydroxy-steroid-Δ^24^-reductase (24-DHCR) catalyzes the conversion of desmosterol into cholesterol (last step of the Bloch pathway) by reducing unsaturation at the 24^th^ position in the flexible alkyl side chain of desmosterol.

As mentioned earlier, cholesterol biosynthesis has been shown to be essential for mammalian cell proliferation [2]–[4]. It has been previously shown that statins block cell proliferation by arresting cells in G1 phase [8], [9]. Statins are competitive inhibitors of HMG-CoA reductase, the key enzyme in cholesterol biosynthesis and are extensively used as cholesterol lowering drugs to treat hypercholesterolemia and dyslipidemia [10]–[12]. Statins represent one of the best selling drugs globally and in clinical history.

In this work, we explored the stringency of the requirement of cholesterol in the regulation of cell cycle progression. In order to achieve this, we employed distal (AY 9944 and triparanol) and proximal (lovastatin) inhibitors of cholesterol biosynthesis. Our results show that cholesterol content increases in S phase and inhibition of cholesterol biosynthesis by triparanol or lovastatin results in cell cycle arrest in G1 phase. Importantly, G1 arrest mediated by lovastatin or triparanol is restored upon metabolic replenishment with cholesterol. These results therefore show that cholesterol, and not even its immediate biosynthetic precursors differing with cholesterol merely in a double bond, is necessary for G1 to S transition during cell cycle progression.

## Materials and Methods

### Materials

Cholesterol, 1,2-dimyristoyl-*sn*-glycero-3-phosphocholine (DMPC), AY 9944, EDTA, Hoechst 33342, penicillin, propidium iodide, streptomycin, gentamycin sulfate, Nile Red, polyethylenimine, PMSF, RNase A, sodium bicarbonate, Tris, triparanol, trypsin and Na_2_HPO_4_ were obtained from Sigma Chemical Co. (St. Louis, MO). DMEM (Dulbecco’s Modified Eagle Medium) and fetal calf serum (FCS) were from Invitrogen Life Technologies (Carlsbad, CA). Lovastatin was obtained from Calbiochem (San Diego, CA). Amplex Red cholesterol assay kit was from Molecular Probes (Eugene, OR). All other chemicals and solvents used were of the highest available purity. Water was purified through a Millipore (Bedford, MA) Milli-Q system and used throughout.

### Cell Culture and Sorting of Cells in Different Phases of Cell Cycle

Rat fibroblast F111 cells were maintained in DMEM supplemented with 3.7 g/l of sodium bicarbonate, 10% fetal calf serum, 60 µg/ml penicillin, 50 µg/ml streptomycin, and 50 µg/ml gentamycin sulfate (complete DMEM) in a humidified atmosphere with 5% CO_2_ at 37°C. This normal fibroblast cell line was previously developed from skin fibroblast cultures of Fisher rats and is a gift from the laboratory of Dr. Thomas L. Benjamin (Department of Pathology, Harvard Medical School). It is also listed in the ATCC catalog. We have earlier reported flow cytometric properties of this cell line [13], [14]. Confluent cells were harvested with 0.1% trypsin-EDTA, suspended in phosphate buffered saline (PBS) with 2% serum and spun down at 300×g for 5 min. Cells were washed once with PBS and resuspended in DMEM supplemented with 3.7 g/l of sodium bicarbonate (plain DMEM) at a concentration ∼10^6^ cells/ml. For labeling, cell suspension was then incubated with 5 µg/ml Hoechst 33342 for 45 min at 37°C. The stock solution of Hoechst 33342 was prepared in water. Hoechst 33342 is extensively used to quantify DNA in live cells. After labeling, cells were sorted using MoFlo (Dako Cytomation, Fort Collins, CO) in G1, S and G2 phases based on DNA content as indicated by Hoechst 33342 fluorescence. DNA bound Hoechst 33342 was excited with 351 nm and fluorescence was collected using 450/65 nm bandpass filter. In order to distinguish single cells from multiplets, “pulse processing” protocol was used and fluorescence from multiplets of cells was gated out using pulse width and fluorescence-area (Fl-A) display.

### Estimation of Total Cellular Cholesterol and Phospholipid Contents

For total cellular lipid estimation, lysate of sorted cells was prepared in buffer containing 10 mM Tris, 5 mM EDTA, 0.1 mM PMSF, pH 7.4. Total cholesterol content of F111 cells sorted in G1, S and G2 phases of cell cycle was estimated using the Amplex Red cholesterol assay kit [15]. Total phospholipid content in sorted cells was determined after total digestion with perchloric acid as described previously [16] using Na_2_HPO_4_ as a standard. DMPC was used as an internal standard to assess lipid digestion. Samples without perchloric acid digestion produced negligible readings.

### Treatment of F111 Cells with Metabolic Inhibitors of Cholesterol Biosynthesis

The stock solution of lovastatin was prepared as described previously [8]. Stock solutions of triparanol and AY 9944 were prepared in DMSO and water, respectively. Cells were grown for 24 h in complete DMEM and then treated with various concentrations of inhibitors for 48 h in complete DMEM. Control cells were grown under similar conditions without inhibitors.

### Metabolic Replenishment of Cholesterol with Serum

After treatment with 2.5 µM lovastatin or 7.5 µM triparanol for 48 h, metabolic replenishment of cholesterol with serum was carried out in two ways. In the first case, treated cells were washed once with PBS and grown in complete DMEM for 24 h. In the second case, treated cells were washed once with PBS and grown in complete DMEM supplemented with additional 10% serum (total 20% serum) both in presence and absence of inhibitors for 24 h.

### Flow Cytometric Analysis of F111 Cells

After treatment with inhibitors, cells were harvested using 0.1% trypsin/EDTA, suspended in PBS and spun down for 5 min at 300×g. Cells were resuspended in PBS with 2% serum, fixed with cold 70% ethanol and spun down for 5 min at 600×g. Fixed cells were washed twice with PBS and labeled with 50 µg/ml propidium iodide for 1 h at 4°C in the presence of 200 µg/ml RNase A in PBS containing 2% serum. Propidium iodide is a well known DNA binding fluorescent dye and is used for quantitating cellular DNA content in fixed cells. Distribution of treated cells in different phases of cell cycle was monitored by FACS Calibur flow cytometer (BD Biosciences, USA) using propidium iodide fluorescence. Propidium iodide was excited with 488 nm and its fluorescence was collected using 585/40 nm bandpass filter. Appropriate gating was used to exclude multiplets of cells from analysis (see Fig. 1A) and 10,000 events were recorded for each experiment. Representative flow cytometry histogram of fixed F111 cells upon propidium iodide labeling with a time-scaled diagram of different phases of cell cycle as an inset is shown in Fig. 1B.

### Visualization of Lipid Droplets in F111 Cells

Cells were grown on Lab-Tek chambered coverglass (Nunc) in complete DMEM for 24 h. For visualization of lipid droplets, cells were labeled with 0.5 µg/ml Nile Red in plain DMEM for 15 min at 37°C and washed twice with PBS. After labeling, confocal imaging of cells was performed in PBS containing 1 mM CaCl_2_ and 0.5 mM MgCl_2_ (pH 7.4). Dual color fluorescence images of cells labeled with Nile Red were acquired at room temperature (∼23°C) on an inverted Zeiss (Jena, Germany) LSM 510 Meta confocal microscope, with a 63×, 1.2 NA water immersion objective under one Airy pinhole. For visualization of neutral lipids, Nile Red was excited with 488 nm, and fluorescence was collected using 505–570 nm bandpass filter. On the other hand, Nile Red was excited with 594 nm and fluorescence was collected using 650–710 nm bandpass filter to visualize polar lipids.

### Estimation of Neutral Lipids in F111 Cells Using Flow Cytometry

Confluent cells were harvested with 0.1% trypsin/EDTA, suspended in PBS with 2% serum and spun down at 300×g for 5 min. Cells were washed once with PBS and resuspended in plain DMEM. Cells were first labeled with 5 µg/ml Hoechst 33342 for 45 min at 37°C, washed once with PBS and then labeled with 0.5 µg/ml Nile Red for 15 min at 37°C. Labeling of cells with Hoechst 33342 was performed to identify cells in different phases of cell cycle. Nile Red is a specific fluorescent probe that is employed for quantifying the intracellular lipid contents by flow cytometry in mammalian cells [17]. Labeling of cells was performed in plain DMEM at a concentration ∼10^6^ cells/ml. After labeling, cells were washed twice with PBS and resuspended in PBS with 2% serum for flow cytometric analysis. The stock solution of Nile Red was prepared in DMSO. Equal amount of DMSO was added to control (unlabeled) cells. Neutral lipids were quantified in G1, S and G2 phases of cells with MoFlo (DakoCytomation, Fort Collins, CO) using Nile Red fluorescence. For this, Nile Red was excited with 488 nm and its fluorescence was collected using 570/20 nm bandpass filter.

### Analysis of Data

Distribution of cells in G1 (blue), S (red) and G2 (green) phases of cell cycle is analyzed by FlowJo version 7.6.1 under appropriate constraints employing Dean-Jett-Fox model [18], [19]. Profiles of Nile Red labeling of cells were analyzed by Summit v4 software. Further plotting and analysis of data were done using Origin software version 6.0 (OriginLab Corp., Northampton, MA, USA) and Microsoft Excel 2007.

### Statistical Analysis

Significance levels were estimated using Student’s two-tailed unpaired *t*-test using Graphpad Prism software version 4.0 (San Diego, CA).

## Results

### Cell Size and Cellular Lipid Content Vary with Cell Cycle Progression

**Figure 3:**
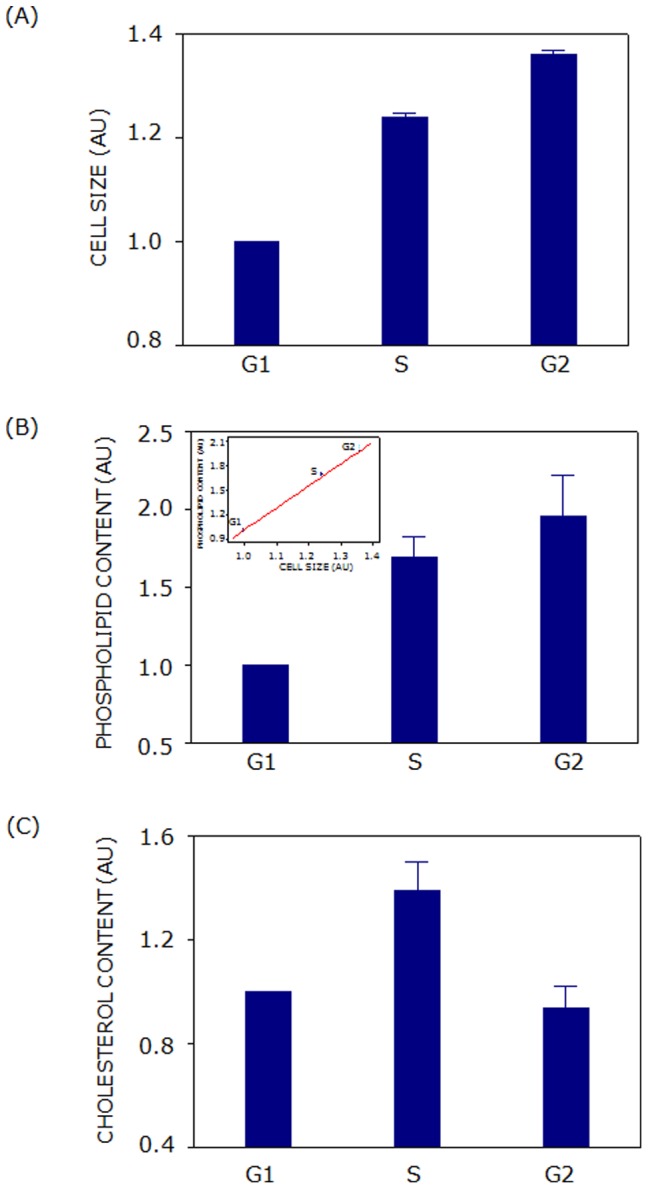
Cell size and cellular lipid content vary with cell cycle progression. (**A**) Cell size increased linearly as cells progressed from G1 to G2 *via* S phase of cell cycle. Total cellular phospholipid and cholesterol contents in G1, S and G2 phases of cell cycle are shown in panels (B) and (C), respectively. Phospholipid content showed an excellent correlation with the cell size in respective phases (shown as an inset of panel (B)). Values are normalized to that of G1 phase. Data represent means ± SE of at least four independent experiments. See Materials and Methods for more details.

F111 cell line was employed for these studies since it has been established as an appropriate model system for cell cycle studies [13], [14]. For lipid quantification, live F111 cells were stained with Hoechst 33342 and sorted under optimal conditions of flow cytometric resolution after exclusion of dead cells and debris (based on a forward scatter threshold). Forward scatter is a good measure of particle size and was therefore used to estimate relative cell sizes in different phases of cell cycle. Fig. 3A shows that cell size was minimum in G1 phase and it showed an increase by ∼24 and ∼36% in S and G2 phases. Similarly, Fig. 3B shows that total phospholipid content of cells increased by ∼69 and ∼96% in S and G2 phases, respectively. Cell size and total phospholipid content therefore showed a continuous increase with cell cycle progression from G1 to G2 *via* S phase prior to cytokinesis. Phospholipid content of cells showed a good correlation with the cell size in respective phases (see inset of Fig. 3B). Interestingly, free cholesterol content of cells displayed a unique dependence on cell cycle progression. Fig. 3C shows that free cholesterol content of F111 cells increased by ∼40% in S phase which subsequently reduced to the level of G1 in G2 phase. These results indicate that free cholesterol content could be important for cell cycle progression.

### Treatment with Lovastatin or Triparanol Results in G1 Arrest of F111 Cells

**Figure 4:**
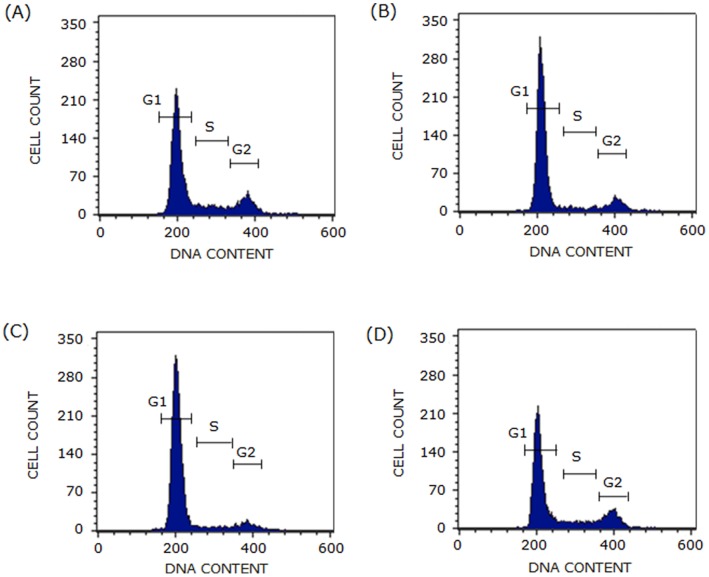
Representative flow cytometry histograms of F111 cells treated with lovastatin, triparanol and AY 9944. F111 cells were treated with inhibitors and fixed with cold ethanol. After fixation, cells were labeled with propidium idodide and analyzed by flow cytometry for their distribution in G1, S and G2 phases. Representative flow cytometry histograms of (A) control cells and cells treated with (B) lovastatin (2.5 µM), (C) triparanol (7.5 µM) and (D) AY 9944 (10 µM) are shown. See Materials and Methods for more details.

**Figure 5:**
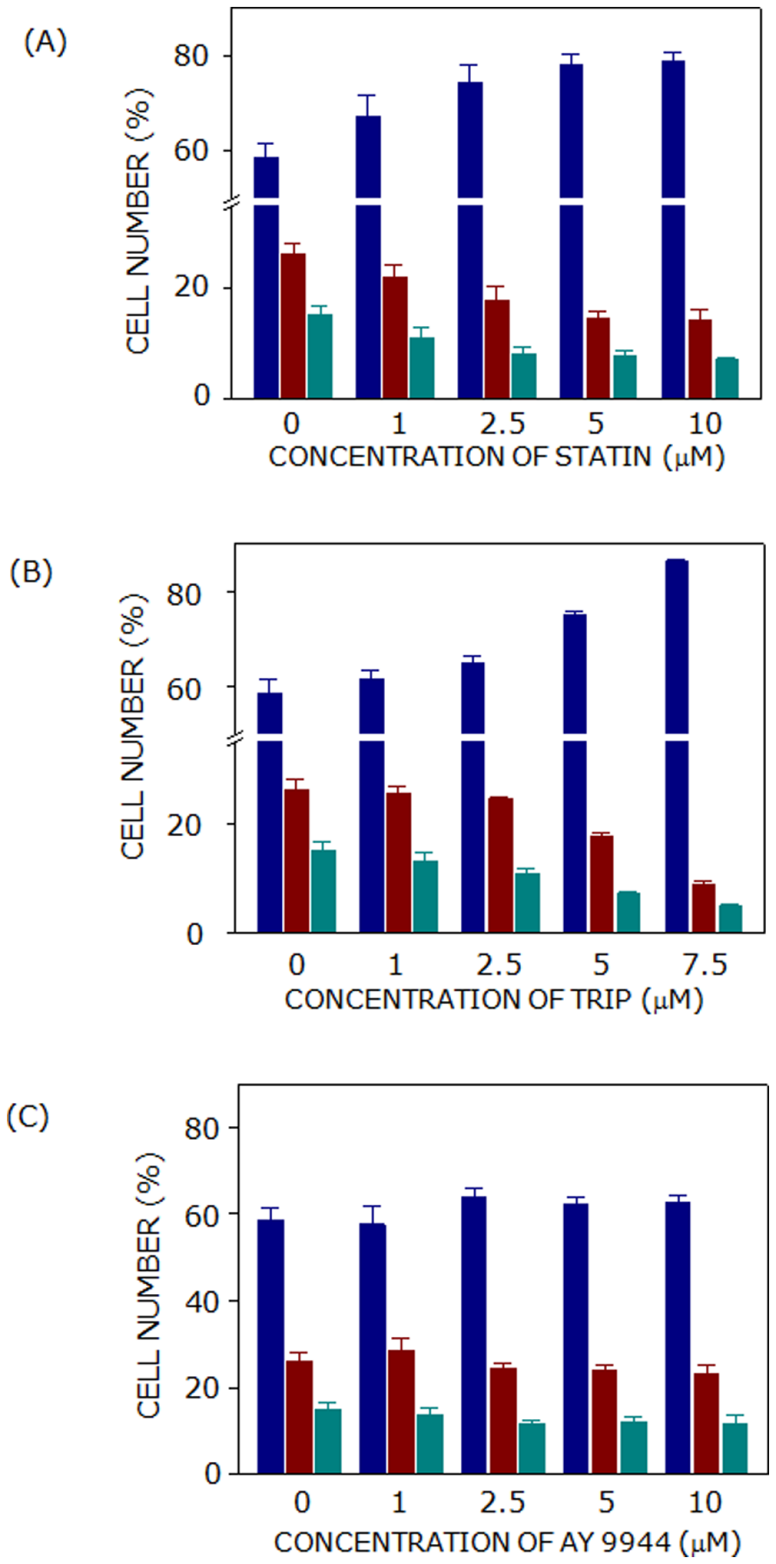
Treatment with lovastatin or triparanol results in G1 arrest of F111 cells. Cells treated with either (A) lovastatin (statin) or (B) triparanol (trip) showed an increase in the number of cells in G1 phase (blue bars) with increasing concentrations of inhibitors. On the other hand, AY 9944 treatment did not affect the distribution of cells in different phases of cell cycle (shown in panel C). Cell numbers in S and G2 phases are represented by maroon and cyan bars, respectively. Data represent means ± SE of at least four independent experiments. See Materials and Methods for more details.

As mentioned earlier, cholesterol biosynthesis in cells occurs by two pathways, *i.e.*, the Kandutsch-Russell and Bloch pathway (see Fig. 2). With the goal of exploring the stringency of cholesterol requirement for cell cycle progression, proximal (lovastatin) and distal (triparanol and AY 9944) inhibitors of cholesterol biosynthesis were employed. Lovastatin is a commonly used statin which lowers cholesterol content by inhibiting HMG-CoA reductase activity. On the other hand, AY 9944 and triparanol result in accumulation of 7-DHC [20] and desmosterol [21], respectively, along with the reduction in cholesterol content. Fig. 4 shows typical flow cytometry histograms (cytograms) for cells treated with lovastatin, triparanol, and AY 9944. Lovastatin and triparanol treatment of F111 cells resulted in cell cycle arrest in G1 phase. In order to estimate the effect of inhibitors in a concentration-dependent manner, cell cycle distribution in G1, S and G2 phases was determined with increasing concentrations of inhibitors. Fig. 5A shows that distribution of control cells in G1, S and G2 phases was ∼59, ∼26 and ∼15%, respectively (details in Table 1). Lovastatin treatment resulted in G1 arrest such that cell numbers increased to ∼74% at 2.5 µM lovastatin (see Fig. 5A and Table 1). The effect of lovastatin on G1 arrest plateaus off beyond 2.5 µM. Similarly, triparanol treatment resulted in cell cycle arrest in G1 phase. Cells displayed progressive arrest in G1 phase with increasing concentrations of triparanol (shown in Fig. 5B and Table 1). Cells in G1 phase were arrested to ∼86% upon treatment with 7.5 µM triparanol. On the other hand, Fig. 5C shows that AY 9944 did not result in any significant change in the distribution of cells in G1, S and G2 phases of cell cycle (Table 1).

**Table 1:** Distribution of cells in G1, S and G2 phases of cell cycle under different treatment conditions.

	Percentage of cells ± SE in different phases of cell cycle		
Condition	G1	S	G2
**Treatment for 48 h**			
Control	58.7±2.8	26.2±2.0	15.1±1.6
Lovastatin (1 µM)	67.2±4.4	21.8±2.5	11.0±1.9
Lovastatin (2.5 µM)	74.3±3.6	17.7±2.4	8.0±1.3
Lovastatin (5 µM)	77.8±2.3	14.5±1.4	7.7±0.9
Lovastatin (10 µM)	78.6±1.8	14.3±1.6	7.1±0.2
Triparanol (1 µM)	61.4±2.2	25.4±1.3	13.2±1.5
Triparanol (2.5 µM)	64.8±1.6	24.4±0.6	10.8±1.1
Triparanol (5 µM)	74.9±0.9	17.7±0.6	7.4±0.4
Triparanol (7.5 µM)	86.1±0.5	9.0±0.5	5.0±0.2
AY 9944 (1 µM)	57.7±4.1	28.4±2.9	13.9±1.3
AY 9944 (2.5 µM)	64.0±2.0	24.3±1.1	11.7±0.9
AY 9944 (5 µM)	63.2±1.7	24.4±1.3	12.4±1.1
AY 9944 (10 µM)	64.1±1.6	23.9±1.8	12.0±1.9
AY 9944 & Triparanol (1 µM)	63.6±0.9	23.7±1.4	12.7±1.0
AY 9944 & Triparanol (2.5 µM)	65.2±1.0	23.5±1.5	11.3±0.8
AY 9944 & Triparanol (10 µM)	75.2±1.8	17.9±1.6	7.0±0.6

### Lovastatin or Triparanol does not Alter Cell Cycle Distribution in the Presence of Additional Serum Cholesterol

**Figure 6:**
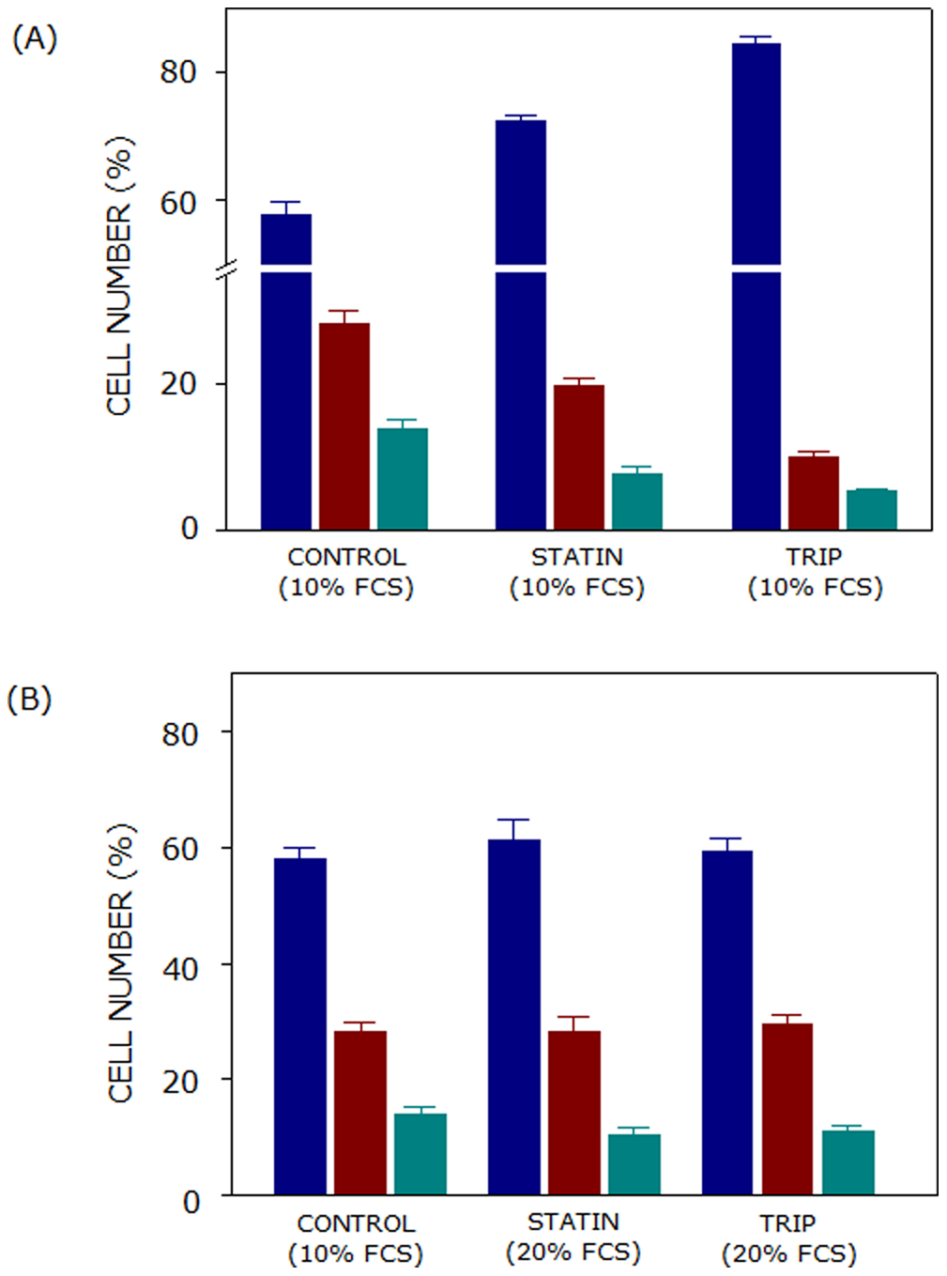
Lovastatin or triparanol does not alter cell cycle distribution in the presence of additional serum cholesterol. Both lovastatin (2.5 µM) and triparanol (7.5 µM) treatment of F111 cells resulted in the arrest of cells in G1 phase (blue bars) of cell cycle in presence of 10% serum (shown in panel (A)). Interestingly, presence of additional serum cholesterol (*i.e.,* DMEM supplemented with 20% serum) abolished the G1 arrest of cells during cell cycle progression (see panel (B)). Cell numbers in S and G2 phases are represented by maroon and cyan bars, respectively. Values represent means ± SE of at least four independent experiments. See Materials and Methods for more details.

Lovastatin and triparanol are known to lower cholesterol by inhibiting cholesterol biosynthesis at proximal and distal ends. Lovastatin or triparanol treatment resulted in cell cycle arrest in G1 phase (Fig. 5A and B). In order to monitor the effect of additional cholesterol on lovastatin and triparanol mediated G1 arrest, F111 cells were treated with inhibitors in the presence of 20% serum cholesterol. Serum (LDL) cholesterol has been shown an effective method for replenishing cholesterol metabolically [20], [22]. Fig. 6A shows that treatment of F111 cells with 2.5 µM lovastatin or 7.5 µM triparanol in the presence of 10% serum resulted in cell cycle arrest by causing accumulation of ∼72 and ∼85% cells in G1 phase, respectively, as compared to ∼58% cells in G1 phase in control population (see Table 1). Interestingly, treatment with 2.5 µM lovastatin or 7.5 µM triparanol in the presence of 20% serum (*i.e.,* DMEM supplemented with 20% serum) did not significantly alter cell cycle distribution of F111 cells (see Fig. 6B and Table 2). This shows that additional serum was able to provide sufficient cholesterol required for cell cycle progression, even when cholesterol biosynthesis was inhibited by lovastatin or triparanol. These observations show that cholesterol requirement, independent of the source (*i.e.,* exogenous or biosynthesis), is crucial for cell cycle progression. In addition, these results indicate that cholesterol, not the intermediates produced during its biosynthesis, is the prime requirement for cell cycle progression in F111 cells.

**Table 2:** Distribution of cells in G1, S and G2 phases of cell cycle under different treatment conditions.

	Percentage of cells ± SE in different phases of cell cycle		
Condition	G1	S	G2
**1. Treatment for 48 h**			
Control (10% FCS)	57.9±2.0	28.3±1.6	13.8±1.4
Control (20% FCS)	51.8±2.1	36.1±2.6	12.1±0.6
Lovastatin (10% FCS)	72.5±1.0	19.7±1.0	7.8±0.8
Lovastatin (20% FCS)	61.3±3.5	28.3±2.5	10.3±1.2
Triparanol (10% FCS)	84.6±1.0	10.0±0.6	5.4±0.4
Triparanol (20% FCS)	59.4±2.2	29.6±1.5	11.1±0.8
**2. Treatment for 72 h (48+24 h)**			
Control (10% FCS)	61.3±1.4	27.8±1.3	10.9±1.1
Control (10% FCS +20% FCS)	49.2±1.6	37.9±1.5	12.9±0.2
Lovastatin	71.1±1.0	19.2±1.6	9.6±0.7
Lovastatin+(10% FCS)	53.2±2.2	32.1±1.0	14.7±1.3
Lovastatin+(20% FCS)	54.0±2.7	29.4±1.9	16.6±1.4
Lovastatin+(20% FCS) & Lovastatin	58.6±2.8	27.7±1.3	13.8±1.7
Triparanol	77.8±0.7	14.4±0.6	7.8±0.7
Triparanol+(10% FCS)	56.5±1.2	30.3±0.8	13.2±1.0
Triparanol+(20% FCS)	55.4±3.2	31.8±2.7	12.8±1.2
Triparanol+(20% FCS) & Triparanol	57.9±1.3	29.7±1.2	12.4±1.1

* 2.5 µM lovastatin and 7.5 µM triparanol were used for all experiments.

### Metabolic Replenishment of Cholesterol Restores the Cell Cycle Distribution of Lovastatin or Triparanol-treated Cells

**Figure 7:**
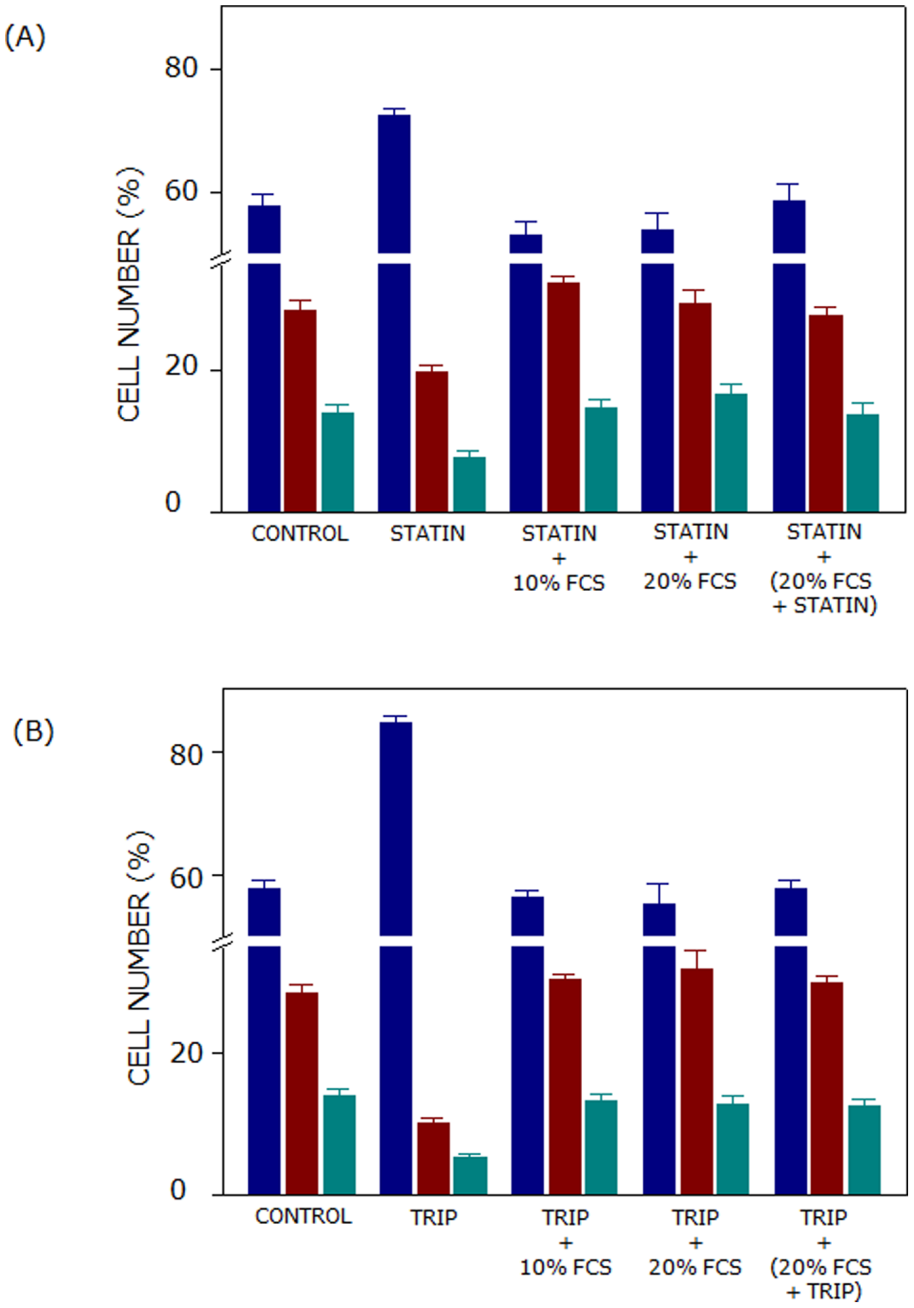
Metabolic replenishment of cholesterol restores the cell cycle distribution of lovastatin or triparanol-treated cells. In order to monitor the reversibility of lovastatin or triparanol treatment on the G1 arrest of cells, we utilized two approaches. In the first approach, cells treated with lovastatin (2.5 µM) or triparanol (7.5 µM) were further grown for 24 h in the presence of either 10 or 20% serum (shown in panels (A) and (B), respectively). In the second approach, cells treated with lovastatin (2.5 µM) or triparanol (7.5 µM) were grown for additional 24 h in 20% serum in the presence of respective inhibitors (see panels (A) and (B)). Cell numbers in G1, S and G2 phases are represented by blue, maroon and cyan bars, respectively. Values represent means ± SE of at least four independent experiments. See Materials and Methods for more details.

In order to examine whether cholesterol replenishment could restore the normal cell cycle distribution, cells treated with lovastatin or triparanol were grown in the presence of serum (LDL) cholesterol. Two approaches were employed to achieve cholesterol replenishment for lovastatin or triparanol-treated cells. In the first approach, lovastatin or triparanol-treated cells were grown for 24 h in the presence of either 10 or 20% serum. Distribution of lovastatin-treated cells in 10 or 20% serum was restored to ∼53 and ∼54% in G1 phase, respectively (see Fig. 7A and Table 2). Similarly, distribution of triparanol-treated cells in 10 and 20% serum conditions was restored to ∼57 and ∼55% in G1 phase, respectively (see Fig. 7B and Table 2). Cell cycle arrest due to inhibition of cholesterol biosynthesis is therefore released upon providing serum cholesterol in medium. In the second case, cell cycle progression of lovastatin or triparanol-treated cells was monitored after growing them for 24 h in 20% serum in the presence of respective inhibitors. Distribution of lovastatin- or triparanol-treated cells in 20% serum cholesterol in the presence of respective inhibitors in the G1 phase was restored to ∼58% (see Figs. 7A and B and Table 2). These results therefore establish that cholesterol is specifically required for G1 to S transition.

### Combined Treatment of AY 9944 and Triparanol does not Show Any Additional (Antagonistic or Synergistic) Effect on Cell Cycle Progression

**Figure 8:**
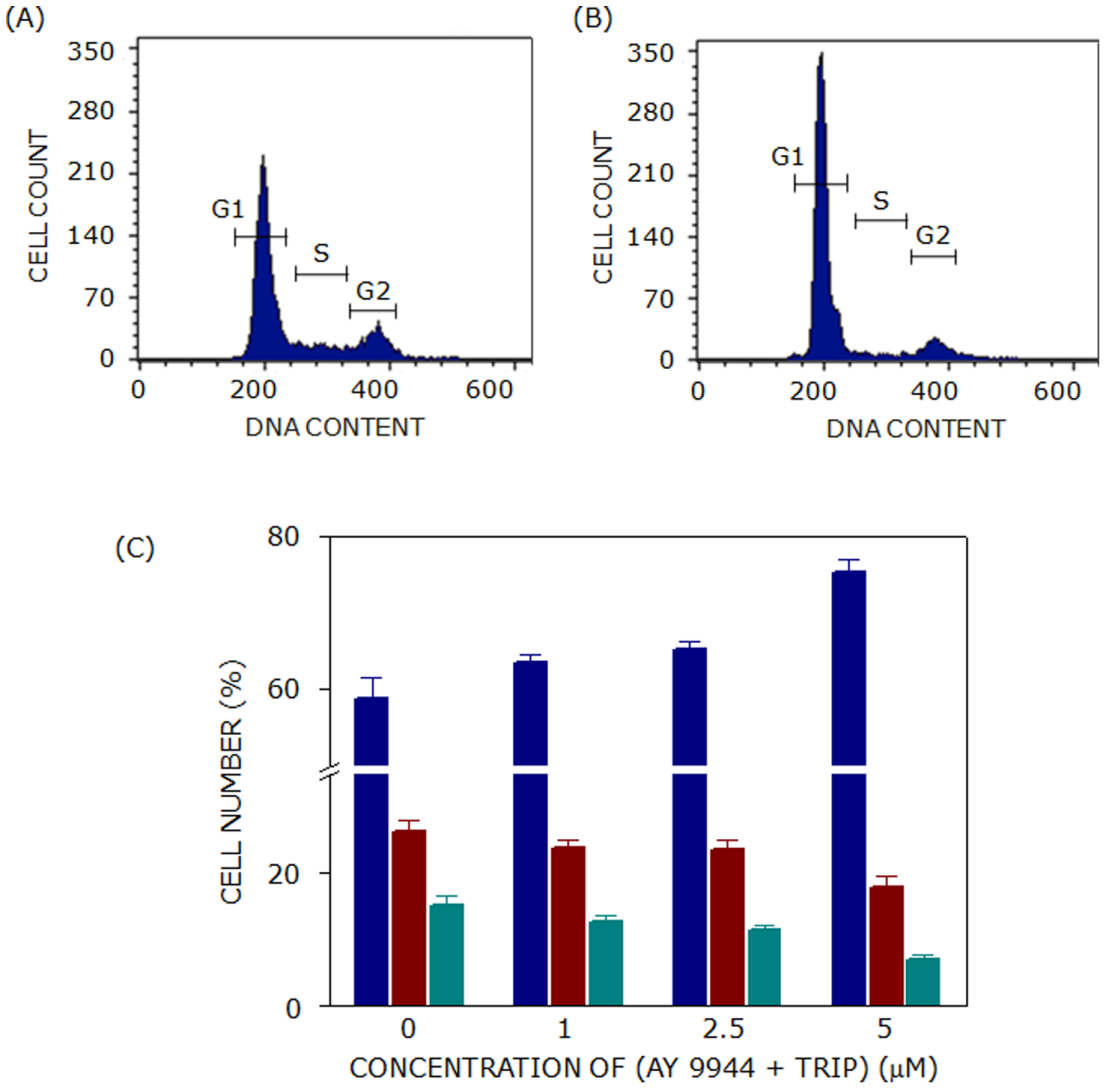
Combined treatment of AY 9944 and triparanol does not show any additional (antagonistic or synergistic) effect on cell cycle progression. Representative flow cytometry histograms of (A) control cells and (B) cells treated with AY 9944 (5 µM) and triparanol (5 µM) are shown. Combined treatment of cells with AY 9944 and triparanol did not result in any synergistic arrest of cells in G1 phase (blue bars) as shown in panel (C). Cell numbers in S and G2 phases are represented by maroon and cyan bars, respectively. Values represent means ± SE of at least four independent experiments. See Materials and Methods for more details.

As mentioned earlier, triparanol and AY 9944 are inhibitors of two different enzymes involved in two pathways of cholesterol biosynthesis (see Fig. 2). In order to explore the cross-talk between these two pathways, the cell cycle distribution of F111 cells was monitored upon treatment with AY 9944 and triparanol in combination. Representative cytogram of cells treated with triparanol and AY 9944 are shown in Fig. 8A and B). The G1 phase contained ∼58% cells in control conditions. Combined treatment (with 5 µM of each of AY 9944 and triparanol) arrested cells to ∼75% in G1 phase which is similar to that of 5 µM triparanol treatment alone (see Fig. 8C and Table 1). Combinatorial treatment of cells therefore did not result in any synergistic or antagonistic effect on cell cycle arrest in G1 phase.

### Neutral Lipid Content Increases with Cell Cycle Progression

**Figure 9:**
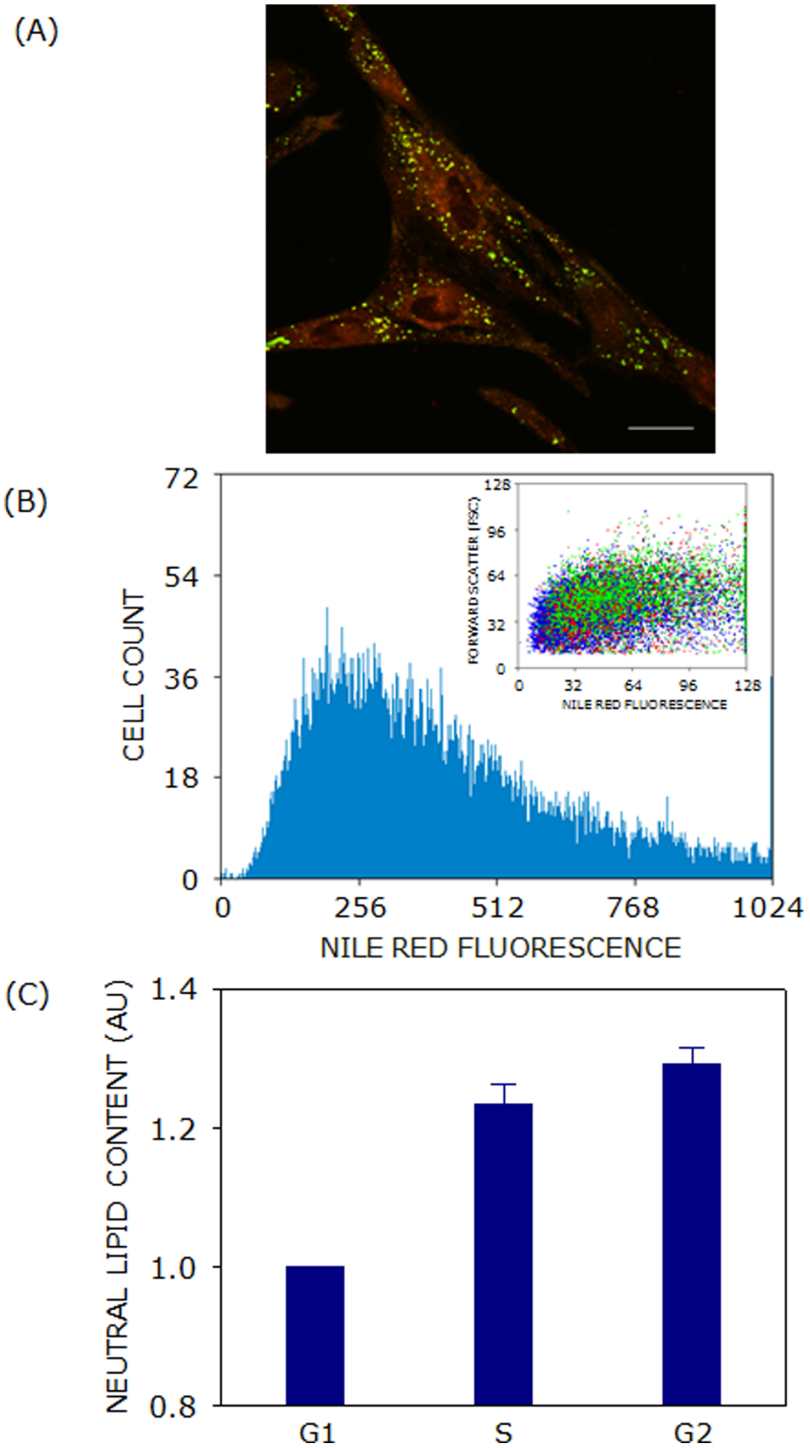
Neutral lipid content increases with cell cycle progression. (A) A representative confocal image shows the presence of neutral (green) and polar (red) lipids in cells, as visualized after labeling with Nile Red. The scale bar represents 20 µm. Neutral lipids in F111 cells were quantified utilizing Nile Red labeling followed by flow cytometric analysis. Typical Nile Red labeling profile of cells is shown in panel (B). A dot plot depicting Nile Red labeling of cells in G1 (blue), S (red) and G2 (green) phases of cell cycle is shown as an inset. (C) Total cellular neutral lipid content demonstrated an increase as cells progressed from G1 to G2 *via* S phase of cell cycle. Data represent means ± SE of at least four independent experiments. See Materials and Methods for more details.

As shown in Fig. 2C, free cholesterol content of F111 cells showed a reduction in G2 phase of cell cycle. Since free cholesterol is known to be esterified and stored in the form of lipid droplets in cells, neutral lipids (lipid droplets) were monitored employing Nile Red in different phases of cell cycle. Nile Red was used for visualization and quantification of lipid droplets in cells [15]. Fig. 9A shows lipid droplets as numerous small green dot-like structures (puncta) in the cell. The total neutral lipid content of G1, S and G2 phases was estimated by flow cytometric analysis of Nile Red and Hoechst 33342 labeled F111 cells [15]. Fig. 9B shows Nile Red labeling and scattering profile (inset) of F111 cells. Neutral lipid content of cells increased by ∼23 and ∼29% in S and G2 phases, respectively as shown in Fig. 9C. The total neutral lipid content therefore showed a significant increase from G1 to S but a marginal increase of ∼5% in G2 phase with cell cycle progression.

## Discussion

In the present work, we explored the role of cholesterol biosynthesis and homeostasis in the regulation of cell cycle progression by employing proximal and distal inhibitors of cholesterol biosynthesis. We observed that cell size and total phospholipid content of cells increased with cell cycle progression from G1 to G2 *via* S phase. Total cellular phospholipid content correlated well with cell size in respective phases, indicating that phospholipids are required as building material for a cell during cell cycle progression. Interestingly, free cholesterol content of F111 cells showed a significant increase in S phase but it reduced in the G2 phase suggesting that cholesterol biosynthesis and cell cycle progression could be mechanistically linked. In order to investigate the relationship between cholesterol biosynthesis and the cell cycle, we monitored cell cycle progression upon inhibiting cholesterol biosynthesis using proximal (lovastatin) and distal (triparanol and AY 9944) inhibitors (see Fig. 2). Lovastatin is a commonly used statin which lowers cholesterol content by inhibiting HMG-CoA reductase activity. On the other hand, AY 9944 and triparanol are well known inhibitors of 7-DHCR and 24-DHCR enzymes, respectively [21], [23]. We observed that treatment with both lovastatin and triparanol resulted in cell cycle arrest in G1 phase. Unlike lovastatin, triparanol does not inhibit the synthesis of non-sterol isoprenoid precursors of cholesterol in F111 cells. These results therefore indicate that cholesterol, and not the non-sterol isoprenoid derivatives, is specifically required for cell cycle progression. Importantly, G1 arrest caused by lovastatin or triparanol treatment was restored upon providing serum cholesterol. In addition, supplementation of additional serum cholesterol rescued G1 arrest of cells even in presence of the inhibitors.

It has been previously reported that non-sterol isoprenoids (mevalonate and its derivatives) are required for cell cycle progression and regulate G1 to S transition in Swiss 3T3 cells [24]–[26]. In contrast to this observation, our results show that inhibition of cholesterol biosynthesis by triparanol at distal end led to G1 arrest. Importantly, this G1 arrest could be restored upon providing additional serum cholesterol to cells. Our results therefore show the specific requirement of cholesterol for G1 to S transition during cell cycle progression. The requirement of cholesterol for cell cycle regulation is rather stringent, as desmosterol, an immediate biosynthetic precursor of cholesterol, was found to be not effective in cell cycle regulation. Lasunción and coworkers previously demonstrated that cholesterol is required for the regulation of cell cycle progression at multiple points in different stages of cell cycle [27]–[30]. In addition, it was shown that some cholesterol analogs did not allow cell cycle progression and arrested cells in G2/M phase [30]. Interestingly, treatment with AY 9944 did not exhibit any change in cell cycle distribution of F111 cells in different phases. These results are in agreement with previous studies where treatment with AY 9944 did not affect cell cycle progression of human promyelocytic HL-60 cells [27]. Notably, we observed a reduction in free cholesterol content of F111 cells in G2 phase of cell cycle. Since free cholesterol is known to be esterified and stored in the form of lipid droplets in cells, we monitored the level of neutral lipids (lipid droplets) in different phases of cell cycle. We observed a marginal increase in neutral lipids in G2 phase of cells.

Modulation of membrane cholesterol content has been shown to affect the function of various membrane proteins [31]–[33]. Interestingly, cell cycle progression was shown to be regulated by PDZ-domain containing proteins [34] which are implicated in the proper localization and clustering of membrane proteins and act as scaffold for various signaling complexes [35]. PDZ-domain containing proteins are believed to interact with underlying actin cytoskeleton. Recently, it has been shown that depletion of membrane cholesterol resulted in the reorganization of actin cytoskeleton [36], [37]. Cellular cholesterol therefore could regulate cell cycle progression by directly influencing the function of membrane proteins involved in cell cycle regulation or indirectly modulating their function through actin cytoskeleton reorganization. Importantly, cholesterol binding has been shown to be crucial for the Hedgehog signaling which is involved in patterning of vertebrate structures such as neural tube, lungs, skin and limbs during embryogenesis [38]. Moreover, various enzymatic defects in the final steps of cholesterol biosynthetic pathway give rise to multiple developmental anomalies owing to impaired Hedgehog signaling [39]. In addition, phosphatidylinositol 3-kinases (PI3-K) and Akt have been recently shown to be essential for Sonic Hedgehog signaling [40]. PI3K/Akt pathway is necessary for cell growth, proliferation and survival [41], [42]. Importantly, PI3K/Akt signaling is shown to regulate sterol regulatory element-binding protein-2 (SREBP-2) pathway [43] which eventually controls the cholesterol biosynthesis and homeostasis in cell [44]. These observations therefore suggest that cholesterol biosynthesis and homeostasis is essential for cell growth and proliferation.

Failure of cholesterol homeostasis causes several health risks and disease states, notably atherosclerosis and Alzheimer’s disease [45]. Importantly, a number of studies have shown that the clinically prescribed cholesterol-lowering drugs, the statins, might reduce the risk of certain cancers [46]–[50]. Moreover, cholesterol level is shown to be modulated along with impairment of cell cycle regulation in cancer [50], [51]. Recently, cholesterol homeostasis has been shown to be restored by LBH589 (panobinostat), an anticancer drug, in NPC1 mutant fibroblasts to normal levels. Panobinostat is an orally available histone deacetylase inhibitor that crosses the blood-brain barrier and is currently in phase III clinical trials for several types of cancer [52]. In the light of these observations, our results could have potential implications for recognizing cholesterol lowering agents such as statin and triparanol as potential anti-tumor drugs. Importantly, defects in cholesterol biosynthesis and homeostasis in the brain have been linked to the development of several neurological disorders such as Alzheimer’s disease, Niemann-Pick type C disease, and Smith-Lemli-Opitz syndrome (SLOS) [6], [20], [53]–[55]. Our results assume broader relevance in the context of diseases associated with cholesterol biosynthesis [56], [57].
